# Phenomenology, Saudi Arabia, and an argument for the standardization of clinical ethics consultation

**DOI:** 10.1186/s13010-021-00099-6

**Published:** 2021-03-12

**Authors:** Abram Brummett, Ruaim Muaygil

**Affiliations:** 1 Department of Foundational Medical Studies, Oakland University William Beaumont School of Medicine, 3331 Squirrel Court, Auburn Hills, MI 48326 USA; 2 College of Medicine, King Saud University and King Saud University Medical City, 6877 Ibrahim Ibn Hadi, Riyadh, 12476 Saudi Arabia

**Keywords:** Clinical ethics consultation, Saudi Arabia, Phenomenology, Credentialing

## Abstract

**Background:**

The purpose of this study is to make a philosophical argument against the phenomenological critique of standardization in clinical ethics. We used the context of clinical ethics in Saudi Arabia to demonstrate the importance of credentialing clinical ethicists.

**Methods:**

Philosophical methods of argumentation and conceptual analysis were used.

**Results:**

We found the phenomenological critique of standardization to be flawed because it relies on a series of false dichotomies.

**Conclusions:**

We concluded that the phenomenological framing of the credentialing debate relies upon two extreme views to be navigated between, not chosen among, in the credentialing of clinical ethicists.

## Introduction

In their critique of standardization in clinical ethics consultation (CEC), Bishop et al. frame the debate as one between pro-credentialing procedural and anti-credentialing phenomenological views [11–13].^1^ The procedural view they describe is committed to quantifying the core aspects of CEC, developing a standardized process for performing CEC, and deploying abstract categories and concepts in consultations in order to transform the consultant into a confident, capable professional. They claim this procedural view is embodied in the Core Competencies Report, and the subsequent development of a Healthcare Ethics Consultant-Certified (HEC-C) program by the American Society of Bioethics and Humanities (ASBH). In stark contrast to the procedural view, they endorse a phenomenological view of CEC that is committed to resisting the quantification of core aspects of CEC, rejecting attempts to standardize the process for performing CEC, and arguing for the bracketing of abstract categories and concepts in consultations in order to create consultants committed to humble inquiry. We will argue that this framing of procedural vs phenomenological views amounts to a false dichotomy between two extreme approaches to CEC. We claim that CEC can be standardized and practiced in a way that navigates *between* these two extreme views. We use experiences from a HEC-C practitioner working in Saudi Arabia to illustrate the importance of standardizing a balanced approach to CEC that avoids the extremes of both procedural and phenomenological views.

## Procedural vs phenomenological approaches to CEC

Bishop et al. frame the credentialing debate as one between procedural vs phenomenological approaches to CEC. Invoking a phenomenological perspective, Bishop et al. write at length on the dangers of imposing our frameworks and procedures on the plenum of reality with all its attendant particularity in ways that can cause one to overlook important features of a consultation. They make this point theoretically by drawing on themes from phenomenologists such as Martin Heidegger with his notion of bringing forth vs challenging forth, and Michel Foucault with his notion of the medical gaze. For Heidegger, the challenging forth mentality seeks to measure, quantify, and control the world, forcing it to conform to one’s purposes. This is set in contrast to a mentality of bringing forth, wherein one seeks to act as a mid-wife, facilitating the means by which being can come into relief as it is, not how we would force it to be ([18], 307–342). For Foucault, the medical gaze refers to the way of seeing that the trained physician brings to her interaction with the patient- a way of seeing that reduces the patient to a kind of matter in motion, a collection of pumps, tubes, chemicals, and lab results, but no longer a person. Describing this process, Bishop et al. write, “the subject carries with him into every place that he goes the set of categories that he deploys. This gaze then can be deployed wherever he goes” ([13], 79). These themes are used to critique the pro-credentialing proceduralistic view they claim is embedded in ASBH’s attempt to standardize CEC.

On their account, proceduralism arises from a desire for recognition from two powerful forces: the practice of medicine and health care institutions, and is dominated by an evidence-based decision-making ideology that speaks the language of quality and efficiency. This ideology seeks to quantify the core elements of CEC into measurable variables (e.g., shortening length of stay, cost savings, alleviating frustrations of medical staff in moral dilemmas) that can be used to demonstrate the usefulness of clinical ethics services ([12], 276–277). The proceduralistic approach is obsessed with the quantifiable goals (e.g., how many consults did our service perform this year), not the goods (e.g., listening, translating, witnessing) of CEC.^2^ In contrast, the phenomenological approach points to “the oxymoron of quantitating qualities,” insisting that the qualitative goods of clinical ethics simply resist easy measurability, and that the goals of medicine (e.g., shorter patient stays) may, at times, conflict with the goods of ethics consultation (e.g., advocating for a patient goal that pursues goods beyond financial efficiency).

For Bishop et al., the proceduralistic ethicist also avoids substantive moral claims and instead focuses on standardizing the *process* of performing an ethics consult—everything from what one does upon receiving a consultation request to developing a standard format for the note that is eventually placed in the patient’s chart. Invoking Tristram Engelhardt, Bishop et al. point out that this purported eschewing of substantive ethical content creates for a confusing equivocation when ethicists “claim normative expertise in matters moral and at the same time deny moral authority in actual cases” ([13], 75–76). They write, “The expertise of the CEC, because she has been formed by a process that avoids substantive content, will be a master of process” ([12], 284).

Instead of asking “What do I need to know?”, the question for the proceduralistic ethicist will be “What is the policy or standard?” [13]. Bishop argues a proceduralistic approach will reify a process of doing CEC (1- what is the question, 2- identify all stakeholders and elicit their views, 3- search for an advance directive or stated preference of the patient, 4- give a specific recommendation) and impose that process in a way that ignores what is particular and local to every consultation. To demonstrate this, he again uses “The Zadeh Scenario” which describes an ethics consultation that did not start at the beginning but rather mid-way through a case, by an ethicist who does not begin by attempting to clearly define the ethical question, skips the search for the patient’s preferences by going straight to the family, and mostly listens and reflects what is said back to the stakeholders without offering a clear recommendation. Bishop notes how this messy reality frustrates the proceduralistic expectations of other commentators, causing some to question whether this was even an ethics consultation ([11], 184–186).

Furthermore, Bishop et al. also take issue with the attempt by Deborah Swiderski et al. to develop a QI tool for standardizing and improving the quality of the ethics consultation note that is placed in the chart. By defining what counts as a quality consultation note and imposing that standard over the practice of CEC, Bishop et al. claim such a QI tool creates a self-referential “epistemological circuit,” whereby “The expert designs the instrument and the instrument assures the expert that he is in fact expert” ([13], 81).

Bishop et al. also argue that proceduralism objectionably deploys a framework on CEC by focusing on abstractions by which a case can be categorized, organized, and prejudged—often prior to the ethicist even engaging with the case, (e.g., “This will be a futility case, I know exactly how to handle these!”).^3^ Bishop et al. reference an exchange between Albert Jonsen and Richard Zaner on this issue. Jonsen recommends a casuistic method for ethics consultation when he writes,

Casuistry will be able to locate the case in a taxonomy of cases, recognize the similarities and differences and appreciate the shift from moral certainty to moral doubt. Above all, casuistic reasoning is prudential reasoning: appreciation of the relationship between paradigm and analogy [i.e., taxonomy], between maxim and circumstance [i.e., morphology], between the greater and less of circumstances as they bear on the claim and rebuttals [i.e., kinetics] ([24], 306).

This casuistic methodology is precisely what Zaner finds objectionable because of the insensitive emphasis on talking over listening, an emphasis that fails to show respect to the person who finds themselves faced with a moral dilemma. Zaner rejects Jonsen’s imposition of abstractions and categories on the clinical encounter, instead seeing the ethics consultant as skilled at helping patients find their “moral voice” with probing questions that give the decision maker room to discover a way forward they can live with ([36], 272). Like Foucault’s critique of the medical gaze with its reductionistic view of the patient, Bishop et al. worry that a procedural approach to CEC will succumb to a similarly reductionistic gaze that closes off aspects of encounters with patients in objectionable ways, attempting to impose our theoretical constructs on clinical realities that will always exceed those constructs ([13], 80).

In short, Bishop et al. worry that a procedural approach with its emphasis on quantifying the core elements of CEC, standardizing the process of consultation, and imposing categories and frameworks for thinking about cases, will transform the consultant from humble inquirer into capable professional ([12], 281). Instead, their phenomenological approach views CEC as a kind of moral inquiry that involves “floundering about in a local, particular situation, a drawing of inferences, a balancing of incommensurable goods, a listening for what is said and unsaid, a traversing across perspectives, a challenging of entrenched positions, and a recognizing of the limitations of languages, shared or unshared” ([12], 290).

## Critique: a framing of false dichotomies

We find that framing the credentialing debate as one between extreme procedural vs phenomenological views relies upon a series of false dichotomies (e.g., process vs content, quantities vs qualities, abstractions vs particularity, confidence vs humility). In contrast, we hold that a more charitable description of the approach to CEC described in the Core Competencies Report is one that avoids the procedural/phenomenological extremes by simply navigating between them. The procedural and phenomenological views may be better understood as opposite ends of a spectrum, not two binary options between which the field of CEC must choose. Furthermore, we hold that the critique by Bishop et al. presumes clinical ethicists to be rather unreflective individuals—so easily enamored by a process, a metric, or a concept that their practice would be overtaken by and reduced to such things. Instead, we hold clinical ethics practitioners to be more intellectually resilient than this, often trained in disciplines that foster reflective mentalities that resist the status quo. We proceed here by identifying a series of false dichotomies in the procedural vs phenomenological framing, and argue there is plenty of conceptual room between these dichotomies within which ethicists can be trusted as professionals capable of using their judgment to navigate. In short, we believe clinical ethics consultants will be able to handle some standardization of their practice without succumbing to the extreme procedural approach to CEC. We conclude the phenomenological critique of credentialing CEC advanced by Bishop et al. is useful in warning of the dangers of standardization but does not amount to a definitive argument against any attempts at standardization*.*

One dichotomy presumed by the procedural vs phenomenological framing is that ethicists will either attempt to quantify everything, even the core, unquantifiable, aspects of CEC, or a phenomenological approach that rejects the quantifying ideology of evidence-based medicine. However, Bishop et al. offer little evidence that ethicists must operate on either of these extremes, and our experience working in ethics services across the United States suggests most services are perfectly capable of quantifying appropriate aspects of their service while not losing sight of the vital importance of the qualitative dimensions. For example, an ethics department in Illinois has published on the aspects of their service that are amenable to quantification [38]. Ethicists employed at that service have not come to regard what can be quantified as the only important parts of consultation practice. In fact, non-quantifiable, core elements of CEC remain central to their service, with one consultant working with the following quote on the wall of her office, “The silence of listening is a form of attention, a gift of self to the other, and a mark of moral generosity. It should manifest an awareness of our humility so as to receive from another person a gift that God is giving us” ([33], 81). An active ethics department in New York has also taken to gathering quantifiable data about their service without losing sight of critical non-quantifiable dimensions of CEC. Senior ethicists at that service have been able to quantify appropriate data while emphasizing the very interpersonal skills (listening, facilitating understanding, and addressing emotional barriers to decision making) that Bishop et al. have worried will be overshadowed by quantifying initiatives [37]. Similarly, at a growing ethics service in California- one where the procedural approach is markedly influential- clinical ethicists are equally invested in enhancing the consultation experience, with all its messy human elements, for patients and practitioners alike. Through non-formal debriefing sessions, consultants attempt to better understand stakeholders’ perceptions of the consultation process in order to better optimize CEC [41]. These examples show that the claim of Bishop et al., that ethicists will attempt to quantify the qualitative dimensions of their practice or come to disregard the qualitative aspects of their service entirely presents an overly pessimistic view of clinical ethicists for which they have offered little evidence and which does not accord with our experience, and, we believe, the experience of most working in CEC.

One may be tempted to read the current method of credentialing (a multiple-choice exam) as evidence that what can be easily quantified and distilled into such an exam will come to be regarded as all that is involved in providing quality ethics consultation. However, the current HEC-C certification exam has this multiple-choice format for budgetary, not ideological reasons. Ethicists have a healthy skepticism towards such an exam accomplishing little more than ensuring a practitioner has a minimum baseline of knowledge and have suggested future iterations of the exam should consider ways to evaluate a practitioner’s *qualitative skills* such as open-ended questions, a portfolio demonstrating more experience, or completion of a formal consultation program under the guidance of an experienced mentor [19]. Again, the point here is that ethicists do not seem tempted to confuse what can be easily quantified with the qualitative aspects that are required to do consultation well.

A second dichotomy presumed by the procedural vs phenomenological framing is that between process and content. It is simply false to describe the Core Competencies Report as presenting a view of standardization that produces ethicists trained *only* in process devoid of content. The facilitation approach to CEC endorsed in the Core Competencies Report calls for mediating the resolution of moral dilemmas *within the range of ethically acceptable options*. Determining what is ethically acceptable presumes ethicists can identify relevant ethical content and thereby make recommendations about what is morally permissible, prohibited, or obligatory in clinical contexts ([21], 7). The fourteen core documents upon which the exam questions are based are full of substantive ethical claims. Consider the following examples, of which there are many more:

1. “Healthcare professionals are not ethically obligated to provide treatments that are not clinically indicated for, or beneficial to, a patient” ([23], 49).
2. “If a patient who lacks capacity strenuously objects to a treatment, the objection should be given ethical weight, and sometimes considerable or even definitive weight” ([23], 11).^4^
3. State laws that provide religious exemptions to child abuse and neglect statutes for parents refusing medical care for children should be overturned ([22], 58).^5^

Process and content need not be thought of as binary approaches to CEC, but both can, and are, incorporated in thoughtful ways by the Core Competencies Report. Furthermore, it is implausible that developing a general process for ethicists to follow when doing consultation will become reified in such a way that ethicists will be unable to think outside that process. Again, we find clinical ethicists to be generally more resilient to such unreflective behavior. Even the authors of the QI tool critiqued by Bishop et al. acknowledge that such a tool should not be reified into a checklist, does not by itself guarantee quality consultation practices, and is merely a good first step towards establishing some consultation standards ([39], 67).

In general, we find that despite having a standard process for decision making (informed consent, advance directive, surrogate, best interests), the academic literature is replete of thoughtful challenges to that process ([29], 69). Consider recent arguments by ethicists that incapacitated patient preferences should have significant, sometimes definitive weight [31], or arguments questioning the usefulness of the living will [15], or thoughtful cases where the standard model should actually be reversed [6]. We do hold that having a general process in place for conducting ethics consultations is helpful but are not convinced this process will be rigidly adhered to by practicing ethicists despite the particularities on the ground.

Interestingly, we find that for all their objections to process, Bishop et al. themselves seem to propose a rather detailed process for performing CEC. They write:

When the clinical ethicist is called, she must explore the various goods as understood by the community of treating physicians. She must explore, as sensitively as possible, why the doctors do not trust the patient’s conception of thriving or what is good for her own life … She must assure that the patient and family understand the medical rationales as articulated by the medical team. She must understand the law, as to what it dictates, and assure that the patient and family understand that more often than not, the law is silent on such issues. She must understand the intermediary role of policy, generally, and also with regard to specific policies that may bear on the patient and medical team. The clinical ethicist is called at times to help negotiate consensus, at other times to challenge the doctor’s understanding of the goods of medicine, or even, dare we say it, to challenge the patient’s understanding of what is medically feasible ([12], 288).

Granted, their process is directed towards discovering goods, but it is a process nonetheless, demonstrating our point that this strict dichotomy between process and content is simply untenable—the two are always interwoven.

A closely related third dichotomy presumed by the procedural vs phenomenological framing is that between ethicists armed with concepts and abstractions that they will deploy in objectionable ways, and ethicists who are able to bracket these considerations and attend to the case in all its radical particularity. We find this dichotomy to also presume an oversimplified binary when the reality is somewhere between both extremes. The debate between Jonsen and Zaner can help illustrate this. Jonsen’s approach involves using analogical reasoning to compare the present case to past cases, and deploying theoretical constructs like best interest, informed consent, or surrogate hierarchies by which to offer humble advice that can “serve as a small blessing in the confusing and conflicted world in which we live” ([25], 437). As we saw above, Zaner critiques Jonsen for focusing on what the ethicist can say to the stakeholders involved as opposed to asking probing questions and listening. Of course, we again see no reason why an ethicist must choose between talking and listening, and regard conscientious ethicists as always searching to find a healthy balance in their practice. When ethicists do deploy abstractions or concepts, we do not see that they do so in a heavy handed way and actively engage in academic debate over the meaning and justification of basic concepts such as autonomy [14, 28], best interests [7], or the harm principle [32], to name a few.

In summary, we find the procedural vs phenomenological framing of the credentialing debate to rely upon a series of false dichotomies that are best understood as tensions to navigate between, not binary options to choose amongst. In what follows, we describe the current state of bioethics in Saudi Arabia, where one of the authors works as a clinical ethics consultant at a major medical center. We use Saudi bioethics practice to highlight the importance of standardizing a balanced approach to CEC in a context that currently falls short of some basic standards in clinical ethics. We invite readers to consider how an anti-credentialing phenomenological approach would serve in such a context.

## Clinical ethics consultation in Saudi Arabia

### The Saudi context: legalistic, scientific, uncredentialed

Saudi Arabian bioethics might appear a curious, perhaps even, inapt choice for an ostensibly American debate on the merits and pitfalls of credentialing clinical ethics consultants. Still, we find that certain characteristics of Saudi bioethical practice acutely illustrate the importance of standardizing the practice of CEC, as standardization serves to establish a baseline of knowledge, skills, and reflective attitudes in practitioners.

The nascent^6^ field of Saudi bioethics has several distinct features. First, its dominant methodology is overwhelmingly legalistic. Saudi bioethics relies heavily on exegeting religious scripture as its underlying moral foundation.^7^ Strict reliance on scripture and subsequent judicial rulings- absent moral analysis, intellectual engagement, or relevant context- stripped Saudi bioethics of the practice’s inherent reflective qualities and rendered it no more than jurisprudence.^8^

Second, Saudi bioethics is unmistakably scientific. Its distinctive presentation as yet another clinical discipline is a direct response to its slow, precarious, and scientist-led entry into a well-established medical field [3]. This measured introduction ensured bioethics displayed the precise, logical, and quantifiable parameters permitting it a space within the technological norms of medicine. It also resulted in the abandonment of bioethics’ intrinsic strengths such as contemplation, deliberation, and conversation. Instead, Saudi bioethics took on the traditional characteristics of medicine and science: hierarchy, paternalism, and inflexibility.

Third, Saudi bioethics is, regrettably, an exclusive domain. Its central practitioners and leading voices are not necessarily those of the most qualified, but rather those of the loudest and the most influential.^9^ Indeed, bioethics remains an elusive discipline to most Saudi healthcare practitioners, a direct result of traditionally cursory bioethics education, an absent intellectual community, and limited scholarship [3]. Unsurprisingly, the curtailment and scientization of bioethics resulted in a detached discipline inattentive to the needs of its own community. We turn now to describe three specific problematic examples of practices in Saudi clinical ethics.

#### Examples of problematic practices in Saudi clinical ethics

##### Spousal consent requirements for medically-indicated hysterectomies and tubal ligations

In Saudi Arabia, the requirement of the husband’s consent for women seeking elective, medically-indicated procedures resulting in irreversible sterility is a widely accepted measure [34].^10^ This prerequisite rests on two ethical claims: protecting the husband’s interest in having children, and upholding men’s legal authority over their women relatives. We find these assertions ethically problematic. Interest in a spouse’s ability to procreate is not always justified; for example, in situations where the couple is legally separated-but not yet divorced- or when women have passed childbearing age. Further, we question the appropriateness of exclusively favoring this interest over a woman’s authority when these procedures represent a viable therapeutic benefit for disabling conditions like chronic pelvic pain, dysfunctional bleeding, or cervical cancer. Additionally, these claims do not invoke a workable plan for when a husband overrides his wife’s consent for a medically indicated procedure. Perhaps most alarmingly, a spouse’s interest in the reproductive abilities of their partner does not appear to extend to women spouses. Few policies requiring women to consent to their husbands’ sterility-producing procedures appear to be in place. Indeed, given this disparity, we find these claims to be supportive of troubling gender discrimination, and needlessly dismissive of a woman’s interest in making independent medical decisions for herself.

These requirements echo legal dictates instructing the male guardianship of Saudi women.^11^ In 2019, however, these mandates were significantly loosened by royal decree [20]. The historic dismantlement of the male guardianship system was noted and celebrated by many. Even so, Saudi bioethicists appear eager to uphold and enact these now obsolete directives through requirements such as spousal consent.

##### ‘Do Not Resuscitate’ guidelines

Spousal consent requirements demonstrate how some ethical claims arrived at by Saudi bioethicists can be infused with certain antiquated and patriarchal ideals, furthering a bioethics that protects some persons’ interests over those of others. Unfortunately, Saudi bioethicists’ examination of resuscitation decisions at the end of life appears similarly ill-considered.

National Do Not Resuscitate (DNR) guidelines are reflective not of careful, bioethical contemplation, but rather of scientific, procedural, and legal considerations. Released by the Saudi Health Council, recommendations are predominately concerned with two areas- the Islamic permissibility of DNR orders, and legal protections for practitioners [35]. For example, recommendations require the consensus of three doctors- a relic of Islamic law in matters of dispute and accountability. By contrast, the document pays little attention to patients’ and/or family members’ involvement in end of life decisions suggesting “communication” with the patient and/or family only when deemed appropriate or necessary by the attending physician, stressing that DNR decisions are ultimately medical decisions that do not require patient and/or family consent [35].

These DNR guidelines are faithfully replicated in the policies of medical institutions all over the country, absent expert ethical examination. Supported by these guidelines, Saudi clinicians have grown accustomed to making decisions unilaterally at the end of life, and vehemently oppose including patients or family members in decision-making. This is made evident in several recent studies including one which discovered that 53.8% of DNR orders in an intensive care unit lacked family involvement [17]. Similarly, another study found that over half of surveyed pediatricians believed that DNR determinations were at the physician’s discretion alone [2]. Alarmingly, DNR guidelines appear inconsistent with recent research on the preferences of Saudi patients. A 2019 study found that almost 70% of interviewed patients wanted to be involved in decisions involving DNR [26], as did another which found that two-thirds of hemodialysis patients wanted to make their own decisions at the end of life [5]. These figures are well illustrated by Al Mutair et al’s important work documenting the lived experiences of family members in an intensive care unit which found that participants deeply valued communication and involvement at the end of a loved one’s life [1].

The focus on legal and Islamic considerations gave the DNR guidelines essential legal and religious validity. The pragmatic approach to ethical issues at the of end of life permitted clinicians a certain comfortable practicality. Still, by allowing bioethics only a nominal presence, fundamental moral claims about life and death were neglected.

##### Informed Consent Guidelines

Saudi bioethicists’ endorsement of the aforementioned DNR guidelines effectively authorized the exclusion of patients and their family members from important end of life discussions. In a similar vein, bioethicists’ support of the Ministry of Health’s (MOH) new informed consent guidelines permits the disregard of patients’ realities and impedes their abilities for self-determination.

Like the DNR recommendations, the MOH’s informed consent guidelines were reached without the input of formally credentialed bioethicists [30]. These guidelines include at least two problematic recommendations. First, the document attempts to identify a formal order of surrogate decision-makers for incapacitated patients. The lexically ordered result favors male relatives over women family members, regardless the degree of closeness [30]. Although the document does not explicitly state it, this order mirrors Islamic rules of inheritance- yet another legal consideration removed from context.^12^ Second, in addressing refusal of treatment, the document states that adults with decision-making capacity may not refuse life-saving treatment [30]. Again, the document provides no ethical support for its determination, although it likely stems from the Islamic, legal maxim on the protection of life. Further, this statement is practically unhelpful; it gives no guidance or moral clarity on just exactly how an adult, capacitated patient might be forced to undergo procedures they have unequivocally declined.

It is disheartening to see pioneering, well-meaning efforts by the Saudi Health Council and the MOH undermined by limited bioethical contemplation. Without substantial input and critical examination by credentialed ethical experts, problematic policies will continue to thrive unchallenged. Absent discernable moral parameters, ethics goes awry. Dubious ethical claims are unquestioned, necessary philosophical conceptions are unexplored, and debates on goods and values are overlooked. Indeed, the absence of intellectual reflection and robust moral consideration results in a stunted bioethical practice incapable of addressing the complex needs of an increasingly sophisticated and diverse Saudi society and risks doing harm to the very population it purports to serve. An underdeveloped, unsophisticated bioethics discipline does more than simply flounder, it categorically fails.

### Conclusions for standardizing CEC in Saudi Arabia

The experience of one of this paper’s authors as a clinical ethicist at a central Saudi hospital finds that ethicists have become little more than tools for enforcing hospital policy such as the ones concerning spousal consent, DNR or informed consent. As uncredentialled ethicists sanction such policies without considered reflection, their endorsement enables ethically questionable policy to continue, and hinders thoughtful examination of its limitations. Indeed, Saudi bioethics’ trepidatious and restricted journey has contributed to the development of an incomplete and ill-formed practice, one which is distinctly theological,^13^ philosophically modest, worryingly exclusive, and seemingly negligent of the multi-disciplinary nature of bioethics. In Saudi Arabia where bioethical practice is still evolving, credentialing and standardization are vital. We hold that Saudi bioethics could be improved through standardization and that applying the phenomenological approach here, with its decidedly anti-credentialing stance and vague insistence on what is “local and particular” to a case, would only serve to maintain the status quo of a practice that causes great harm not only to individual patients and practitioners but to the practice of bioethics itself. In fact, we contend that Bishop et al’s concerns of an unreflective practice, uninformed process, and unfulfilled potential -as levied against the procedural view- are not far from the current realities of Saudi bioethics. Credentialing, in this context, could be a solution to these problems, not the cause of them.

At this juncture, it is perhaps salient to consider the appropriateness of applying ASBH’s credentialing in a context so distinctly different from which it was first envisioned. In truth, the adoption of ASBH’s credentialing process would hardly be a novel occurrence for Saudi healthcare. Since its inception in the 1950s [4], the Saudi healthcare system has often looked to and borrowed from the healthcare procedures, designs, and guidelines of other countries, utilizing these experiences to benchmark its own progress towards a well-developed, world class health care system. In doing so, Saudi practitioners have been careful to alter and innovate in accordance with local customs and norms. Through its implementation of international guidelines, and its substantial number of Western trained Saudi medical professionals, Saudi healthcare became a true reflection of global medicine.

The inclusion of ASBH’s CEC credentialing guidelines is the next natural step for Saudi healthcare. ASBH’s process offers important educational resources, tested methods, and examined intellectual assumptions that could serve as an important anchor for bioethics in Saudi Arabia. Like its clinical predecessors, ASBH’s credentialing standards must be conscious of the local environment. They must be adapted to engage, challenge, and empower local context and tradition- without giving way to ill-advised, ill-considered ethical claims that do more harm than good.

The process of credentialing ensures this happens in two ways. First, through the substantive education of would-be bioethicists in bioethical discourse, methods, and overarching assertions. Second, through the establishment of a designated forum where bioethicists may debate policies and practices. Credentialing imparts substance and contemplation into a potentially unreflective ethical practice while also identifying and excluding untrained and harmful practitioners. Indeed, credentialing makes for a stronger, fairer, and frankly better, Saudi bioethics.

## Conclusion

In this paper, we have described the framing of the credentialing debate by Bishop et al. as one between procedural and phenomenological views. We argued this procedural vs phenomenological framing relies upon a series of false dichotomies (process vs content, quantities vs qualities, abstractions vs particularity, confidence vs humility) that are best understood as tensions a responsible clinical ethicist can navigate between not options she must choose among. The phenomenological critique of CEC is useful for outlining warnings about potential pitfalls in the process of credentialing (such as never reifying processes and standards to such a degree that consultants become closed off to the many complexities of any particular case), but we disagree that a generally anti-credentialing view must follow. We described the practice of CEC in Saudi Arabia to illustrate a context where standardization is especially needed. It is indeed a delicate balance between strict proceduralism and unanchored phenomenology, but we hold the pursuit of such a mean between two extremes to be an achievable good for the responsible practice of CEC.

## Data Availability

Not applicable.

## Abbreviations

CEC: Clinical Ethics Consultation
ASBH: American Society for Bioethics and Humanities
SCFHS: Saudi Commission for Health Specialties
DNR: Do Not Resuscitate
MOH: Ministry of Health

## Footnotes

1 Bishop also critiques attempts to standardize palliative care in ways that are very similar to his critiques of standardization in CEC [8–10]. We hold our critiques of the phenomenological approach in this paper largely extend to Bishop’s arguments in these other works.

2 In one demonstration of the proceduralistic obsession with goals, Bishop notes that none of the commentaries on a clinical ethics consultation (“The Zadeh Scenario”) mentioned goods in their analysis, but only goals, e.g., the goals of care, the ethical goals, etc. ([11], 189).

3 To help make this point, Bishop is fond of quoting Theodor Adorno saying “objects do not go into their concepts without leaving a remainder” ([10], 388). The basic idea from the phenomenological tradition here is, again, that the messy reality of CEC will always exceed our attempts at conceptualization.

4 Ethical considerations for deciding whether to honor an incapacitated refusal include, “the patient’s current level of capacity, the degree to which the refusal is in line with the patient’s previous statements, the burden of treatment, and the expected benefit” ([23], 11).

5 For more examples of specific substantive ethical claims contained in the study materials for the HEC-C exam, see (blinded for review).

6 Bioethics’ first venture into the Saudi Arabian setting occurred in the 1990s as a handful of medical schools introduced perfunctory courses on professionalism and ethics into their curricula Blinded for review. Bioethics’ full integration into the health care system, however, was noticeably delayed as little attention was directed towards cultivating the discipline. In the face of rapid expansion in healthcare infrastructure- one focused primarily on medical research and empirical, evidence-based practice- bioethics was necessarily relegated to a secondary, oft-forgotten, position. Nearly three decades later, amid imminent, significant transformations in the healthcare system [30], and palpable changes in social norms, a renewed interest in bioethics has emerged in Saudi Arabia.

7 Saudi society is deeply influenced by religious tradition. The findings of the Senior Council of Scholars, the country’s highest religious authority, not only play a vital role in the codification of law through religious interpretations and decrees, but also affect social policy and local customs. Saudi bioethicists similarly follow the council’s directives.

8 While determining the legal, Islamic standing of an action is certainly important, it is insufficient as an ethical, moral framework for it does not tell us whether or not an action is *morally* permissible [16]. For example, an Islamic fatwa may deem it legally permissible to discontinue life support based on a physician’s determination of futility. However, mere permissibility does not resolve the multitude of questions and considerations that arise in these cases, for that, a robust, morally rigorous bioethics practice is necessary.

9 An extensive review of Saudi bioethics publications and contributions finds strong dominance of traditionally powerful voices- those of men writing from within a religious tradition- and a troubling absence of the accounts of women and other minority populations. This review includes the Saudi Commission for Health Specialties’ (SCFHS) manuals *Professionalism and Ethics Handbook for Residents: A Practical Guide* and *The Code of Ethics for Healthcare Professionals,* The Saudi Society for Studies in Medical Jurisprudence, and Contemporary Bioethics: Islamic Perspectives by Al-Bar and Chamsi-Pasha. As well as a review of bioethics institutions including King Abdullah International Medical Research Center, King Abdulaziz City for Science and Technology, and The National Committee on Bioethics, and a review of various published literature by Saudi bioethicists [40].

10 The source of this requirement is found in the *Code of Ethics* of the SCFHS the country’s leading authority responsible for the credentialing, certification, and regulation of medical professionals and medical practice [34]. It is also reflected in the policies of many Saudi hospitals, as well as in the practice of the medical community.

11 Under the guardianship system, every woman -regardless of age or competence- had to be under the legal guardianship of a male relative. Women could not travel, open a business, marry, or live independently without the written permission of their “guardian,” often a father or husband but sometimes a brother, an uncle, or even an adult son [20].

12 Islamic rules of inheritance constitute a complex and detailed system that is difficult to dilute into a footnote. What is of relevance here, however, is the division of heirs as outlined in the Quran and Sunna-the sayings and actions of the Prophet Mohammed. Clear Quranic verses establish the first group, ‘*fara’id*’, or those who are entitled to predetermined, non-negotiable shares of the inheritance. After the designated heirs receive their share, the rest is divided amongst the second group ‘*asaba’*, or male agnatic blood relatives. According to the Sunna, male agnates receive an unlimited residuary [27]. After naming the father, grandfather, and mother, the informed consent document names *asaba* relatives in their descending order of appropriate surrogate decision-makers, marginalizing women relatives and their decedents, and disregarding others with close relationships with the patient.

13 The contention is not that bioethicists consulted religious scripture or Islamic scholars but rather that they simply accepted recommendations even if they were clearly problematic or removed from context.
